# The Medico-Social Aspects of the Consumption Problem

**Published:** 1913-04-19

**Authors:** 


### THE MEDICO-SOCIAL ASPECTS OF THE CONSUMPTION PROBLEM.

### II.—The Incidence of Consumption.

It is highly necessary, before discussing the general aspects of the problem of consumption, to have in mind a clear conception of what, in modern times, is meant by the term tuberculosis. From the point of view of the pathologist, tuberculosis means that state of the body in which altered tissue changes result as the consequence of the invasion of a specific bacillus first demonstrated by Koch. This is the tubercle bacillus which belongs to the group of acid-fast bacilli, among which are the bacilli of certain grasses and of leprosy, the latter of which closely resembles in some ways the tubercle bacillus. Outside the body, the bacillus of tuberculosis is very difficult to grow in culture, but it has been grown on blood serum and on potato. In the body its growth may at times be exceedingly rapid if certain conditions are favourable, but in the vast majority of cases it is a slowly growing organism whose vitality is not very high and against which the body tissues display a wonderful and resistive power.

Tuberculosis is, for simplicity of description, divided into two main forms, closed and open tuberculosis. Among the former the various forms of joint and glandular affection are chief; of the latter the most important form is tubercle of the lungs in its later stages. This latter, and not so much what is known as surgical tuberculosis, is the fright of the public; it is against this form that the main efforts of those who attack the disease are specially directed.

### Some Statistics and their Moral.

While statistical evidence, in view of the fact that the question of correct diagnosis is always somewhat in doubt in the returns issued by the Registrar-General, is unsatisfactory in general, it is, so far as this matter of phthisis pulmonalis is concerned, of some value to show whether the disease is increasing or not. Dr. Ogle has paid special attention to the figures, and his tables are interesting, although they must be corrected by later statistics. For example, it is obviously absurd to say that out of every million children under five years of age some sixteen hundred on an average die every year of phthisis; the diagnosis here must be tuberculosis, miliary or generalised tuberculosis being much the most frequent form in children. On the other hand, the statistics for adults are probably correct to a fair degree of accuracy, the mistaken diagnoses being corrected automatically so far as statistics are concerned by those cases which are overlooked. For adults the average annual mortality per million of adult population during the years 1851-80 was 2,423 for both sexes for all ages, the highest mortality being 4,060 for adults between twenty-five and thirty years of age. For male adults the highest figure is 4,097 (thirty-five years of age), and for females 4,165 (twenty-five years of age). The figures given for the ages of ten and fifteen years are obviously over-estimates. Pulmonary phthisis in children under fifteen is a rare disease.

### The Decline of Consumption as a National Disease.

The vital statistics of consumption incidence and mortality must not be taken as beyond criticism. Yet these figures give us some index to the prevalence of disease, and serve to show that in this country at least there has been a steady decline in the mortality rate from pulmonary phthisis, not perhaps a very large decline, but still an appreciable fall. It is noteworthy—and this is a point that our readers should consider very carefully—that this decline does not coincide with the introduction of sanatorium treatment; it is an even more striking fact that a similar decline, of percentage equality, is not shown in the figures of countries such as Germany where sanatorium treatment has been tried more consistently and more generally than with us. The decline is less marked in Scotland than in England; it is much lower in Ireland, again, than in Scotland. What is the cause of such decline? This is a matter on which expert views differ very widely. Some authorities make no secret of their view that the decline is in reality a fictitious fall due to other causes than phthisis being put down on the death certificates. We do not think that this view is justified, for it is absurd to imagine that the fear of tuberculosis is so great with the public that a medical man can bring himself to make a false death declaration with the object of sparing the feelings of relatives.

### Conflicting Explanations.

Another view is that diagnosis is more accurate and that fewer cases of lung complaints are indiscriminately put down as consumption and that this vitiates the value of the statistics. While there is some ground for this supposition, we think that it argues rather on the other side, for we have seen that, in children at least, the medical man is only too prone to diagnose phthisis on insufficient grounds. A glance at the wide variation of figures of incidence of consumption among school children, as given in Sir George Newman's latest report, will show how unreliable is the diagnosis of school doctors who apparently have made a rather closer study of diagnosis than the average practitioner, and who may be supposed to have better diagnostic tests available. It is, therefore, rather far-fetched to ascribe the fall in the mortality figures to greater discrimination in diagnosis. A third view is that sanatorium treatment is responsible for the decline. This view is untenable by anyone who is acquainted with the history of sanatorium treatment, and who takes the trouble to follow the progress of that treatment and its chronicled results; to this aspect of the matter we shall also have to return in a future article. Yet another opinion is that the change is due largely to improved hygienic conditions and sanitation; with this view most authorities agree, and certainly it seems justifiable to attribute to these improvements some of the merit for the decline in the mortality, and therefore in the incidence of consumption in England. The mystery is why there has not been an equally striking fall in Germany, where improved sanitation has been the rule during the last two decades.*

The German Example.

A German authority, Grotjahn, in discussing this side of the matter in his chapter on "Institutions for Sufferers from Lung Complaints," writes as follows:—

"Especially remarkable is the decline in consumption incidence in Prussia. During the decade 1876-86 the mortality figure was comparatively high. From 1886 it steadily decreased year by year, so that in 1906 it had decreased by 30 per cent. Although the population has rapidly increased, it is estimated that at present there are twenty thousand fewer deaths from consumption every year in Prussia than was the case twenty years ago. In Austria and Hungary there has been no decline in the mortality figures. . . It is difficult to say to what such decline must be ascribed. Probably more than one factor combined to produce it. The improvement in the social conditions of the lower classes, and our improved knowledge concerning the dangers of infection, have certainly contributed to achieve this culmination. But I am firmly convinced that the most important factor of all has been the better provision, either at home or in institutions, for the care of patients in advanced stages of the disease."

As a proof of this contention Grotjahn cites the figures for Stockholm, where the decline has been very marked in proportion to the provision made for dealing with advanced cases. We shall have occasion to deal with this point again. For the present we commend its consideration to our readers. One of the main views we shall endeavour to support in these papers is the contention that sanatorium treatment alone is quite inadequate to deal with the problem of consumption, since such treatment holds out no chance for the advanced case. The view we shall put forward is that while everything must be done to help the incipient case, more must be done to deal with the advanced case; it is the latter, not the former, that is chiefly responsible for the spread of the disease, and once we have grasped the fact, lately so forcibly pointed out by Sir George Turner, that the advanced case of phthisis is more dangerous to the community than a case of leprosy, we shall have made a big step in advance.

* A further view is that the decline is due to an acquired racial immunity; we discussed this opinion in a leading article recently when dealing with Dr. Maynard's evidence before the South African Commission.

